# Whole Brain Radiation-Induced Impairments in Learning and Memory Are Time-Sensitive and Reversible by Systemic Hypoxia

**DOI:** 10.1371/journal.pone.0030444

**Published:** 2012-01-18

**Authors:** Junie P. Warrington, Anna Csiszar, Matthew Mitschelen, Yong Woo Lee, William E. Sonntag

**Affiliations:** 1 Oklahoma Center for Neuroscience, University of Oklahoma Health Sciences Center, Oklahoma City, Oklahoma, United States of America; 2 Reynolds Oklahoma Center on Aging, Donald W. Reynolds Department of Geriatric Medicine, University of Oklahoma Health Sciences Center, Oklahoma City, Oklahoma, United States of America; 3 Department of Biomedical Sciences and Pathobiology, School of Biomedical Engineering and Sciences, Virginia Tech, Blacksburg, Virginia, United States of America; Massachusetts General Hospital/Harvard Medical School, United States of America

## Abstract

Whole brain radiation therapy (WBRT) is commonly used for treatment of primary and metastatic brain tumors; however, cognitive impairment occurs in 40–50% of brain tumor survivors. The etiology of the cognitive impairment following WBRT remains elusive. We recently reported that radiation-induced cerebrovascular rarefaction within hippocampal subregions could be completely reversed by systemic hypoxia. However, the effects of this intervention on learning and memory have not been reported. In this study, we assessed the time-course for WBRT-induced impairments in contextual and spatial learning and the capacity of systemic hypoxia to reverse WBRT-induced deficits in spatial memory. A clinical fractionated series of 4.5Gy WBRT was administered to mice twice weekly for 4 weeks, and after various periods of recovery, behavioral analyses were performed. To study the effects of systemic hypoxia, mice were subjected to 11% (hypoxia) or 21% oxygen (normoxia) for 28 days, initiated 1 month after the completion of WBRT. Our results indicate that WBRT induces a transient deficit in contextual learning, disruption of working memory, and progressive impairment of spatial learning. Additionally, systemic hypoxia completely reversed WBRT-induced impairments in learning and these behavioral effects as well as increased vessel density persisted for at least 2 months following hypoxia treatment. Our results provide critical support for the hypothesis that cerebrovascular rarefaction is a key component of cognitive impairment post-WBRT and indicate that processes of learning and memory, once thought to be permanently impaired after WBRT, can be restored.

## Introduction

Close to 1.6 million new cases of cancer [1] and 64,530 primary brain tumors are expected to be diagnosed in the United States in 2011 [2]. The most common form of treatment for metastatic or primary tumors located in brain regions that are difficult to surgically remove continues to be whole brain radiation therapy (WBRT) [3]. Although this treatment regimen is effective in eliminating tumors, damage to normal brain tissue is inevitable. Several studies have shown that cognitive deficits occur in a relatively large percentage of brain tumor survivors, becoming evident months to years after treatment [3]–[6].

Similar to the human studies, impairments in learning and memory have been reported in rats 6–9 months [7] and 12 months [8] after WBRT. Although the specific etiology for the deficits in learning and memory have not been established, previous studies suggest that decreased neurogenesis [9], increases in inflammatory cytokines [10], degradation of extracellular matrix [11] and/or alterations in synaptic morphology [8] may contribute to impaired CNS function. The effects of radiation on many of these processes are highly dependent on dose and radiation regimen, but endothelial cells and vasculature have been shown to be particularly sensitive to the effects of radiation. Endothelial apoptosis [12] and disruption of the blood-brain barrier [13], thickening and vacuolation of the vascular basement membrane [14], and vascular rarefaction [15] have been reported. Although much research remains to be performed, these studies, and others, suggest that WBRT affects multiple pathways and cell types within the CNS ultimately leading to a decline in learning and memory.

Recently, we provided the first information that fractionated WBRT induces a profound capillary rarefaction in hippocampal subregions, brain areas critical for learning and memory, in mice [16]. The vascular rarefaction occurs rapidly and persists despite the presence of local tissue hypoxia suggesting that angiogenic mechanisms within the brain are permanently damaged by WBRT. Nevertheless, when mice are subjected to one month of systemic hypoxia, complete recovery of hippocampal capillary density occurs. Because of the importance of an adequate vascular supply for normal function of the central nervous system (CNS), we reasoned that WBRT-induced vascular rarefaction could be a key factor contributing to the deficits in learning and memory and that the restoration of cerebrovascular density in response to systemic hypoxia may result in improved cognitive function.

The aim of this study was to establish the time-course for the induction of cognitive deficits following the administration of a clinically relevant regimen of WBRT using two different cognitive domains, and determine whether systemic hypoxia can reverse WBRT-induced cognitive impairments. We report that following WBRT, significant early impairments in contextual learning are evident but these deficits are transient and recover by 3 months post-treatment. On the contrary, spatial learning becomes progressively more impaired with time. Importantly, we demonstrate that systemic hypoxia reverses WBRT-induced deficits in spatial learning and vascular density, and that these improvements are maintained for at least 2 months post-hypoxia. This is the first study, to our knowledge, that reports a time-dependent analysis of cognitive function following a fractionated series of WBRT in mice using multiple cognitive domains and demonstrates a complete reversal of learning impairments with systemic hypoxia.

## Results

### WBRT induces early transient deficits in contextual learning

**Figure 1:**
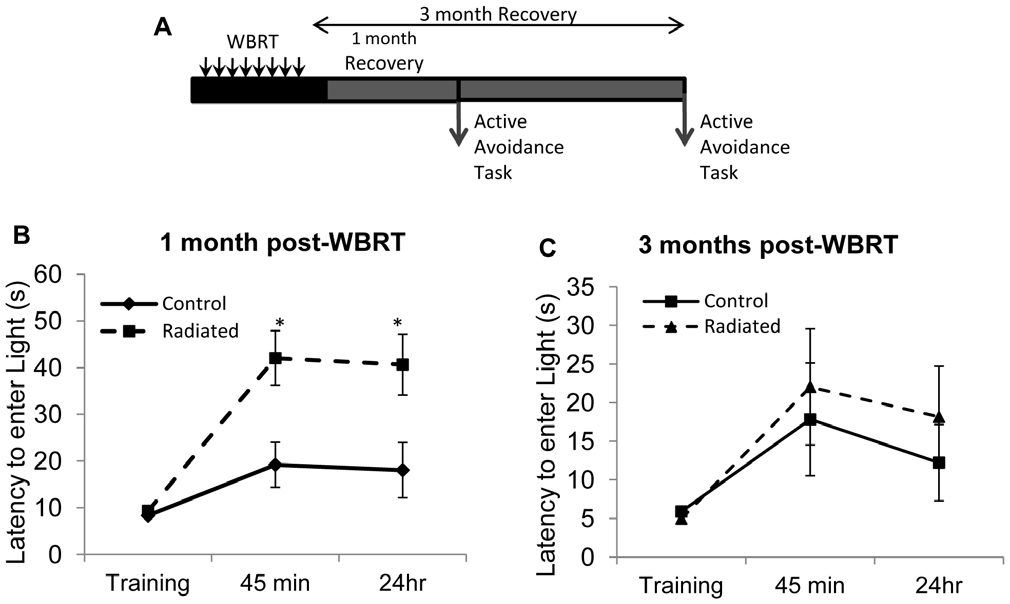
Transient cognitive deficits in contextual learning. (A) Schematic of the experimental design used. (B) At 1 month post treatment, performance of radiated animals was significantly impaired compared to controls at 45 min and 24 h following training. (C) Recovery of learning and memory function was evident at 3 months post-WBRT. Data represent Mean± SEM. *p<0.0167 compared to controls.

In the active avoidance task, mice were trained to form an association between a foot-shock and the dark chamber of a 2-chambered box at 1 or 3 months post-WBRT (Figure 1A). At 1 month post-WBRT, radiated animals required a significantly longer time to escape to the light chamber [F(1, 93) = 16.05; p = 0.0001]. Additionally, there was a significant Group × Time interaction between training and time of testing [F(2, 93) = 3.5; p = 0.0341]. During the training period, there were no differences between the control and radiated groups in the latency to escape to the lit chamber (9.37±0.31s vs. 8.36±0.35s (p = 1.000; Figure 1B). One month after radiation, the mean latency during the 45 min test period for the non-radiated group was 19.21±4.81s while the latency for the radiated group was 42.03±5.87s (p = 0.0143). Memory was reassessed 24 h after training and the radiated animals continued to exhibit deficits in memory, requiring more time to escape to the lit chamber compared to the control animals (40.64±6.52 vs. 18.07±5.9s, p = 0.0162).

To avoid potential confounding effects of repeated testing, a separate cohort of mice was tested on the active avoidance task three (3) months post-radiation (Figure 1C). At this time point, there were no significant effects of radiation on performance [F(1, 73) = 0.20; p = 0.6571]. No differences were observed between groups during the training trials, at 45 min or 24 h post-training (p>0.05) indicating recovery of contextual learning 3 months following fractionated WBRT. Interestingly, 1 month following training, both groups demonstrated retention of the task (data not shown) providing evidence that once animals have mastered the task, ability for recall persists.

### Radiated animals display deficits in spatial learning two months post-treatment

**Figure 2:**
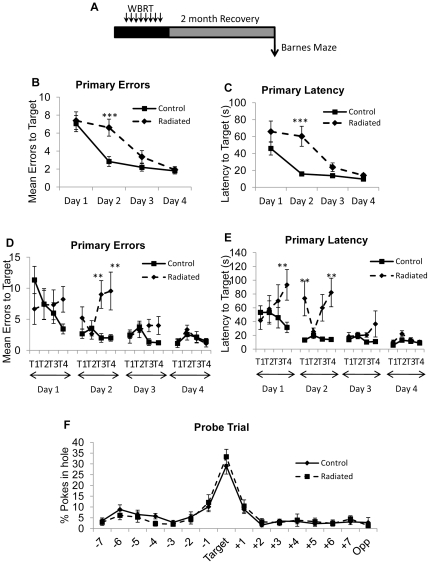
WBRT-induced learning deficits in spatial learning are evident at 2 months. (A) Mice were subjected to fractionated WBRT and allowed to recover for 2 months, after which animals were tested in the Barnes maze. Radiated mice showed deficits in learning acquisition as measured by (B) primary errors and (C) primary latency. Performance on the Barnes maze represented as primary errors (D) and primary latency (E) over trials. No differences in memory retention (Day 10 Probe trial) (F). Data Represent Mean ± SEM. N = 9 per group. **p<0.0031; ***p<0.0001 compared to controls.

Spatial learning was assessed in a separate cohort of mice 2 months post-WBRT (Figure 2A). Mice were trained to use visual cues in the Barnes maze to locate a target box, hidden beneath one of the 16 holes in a circular maze. Four training trials per day, with an inter-trial interval of 20 min, were conducted over 4 days. The number of errors (Primary Error) and time to locate the target hole (Primary Latency) were recorded. The number of primary errors [F(1, 256) = 7.53; p = 0.0065] and primary latency [F(1, 256)  = 23.39; p<0.0001] was significantly higher in the radiated group compared to the controls . Subsequent analysis of primary errors revealed a significant interaction between Group × Day of training [F(3, 256)  = 3.10; p = 0.0274] and Group × Trial [F(3, 256)  = 4.13; p = 0.007]. Animals in the radiated group demonstrated significant impairments in acquisition on day 2 of training making more errors (Figure 2B; p = 0.0031) and requiring more time to reach the target hole compared to controls (Figure 2C; p<0.0001). Further analysis of primary errors for individual trials revealed that trials 3 and 4 on day 2 contributed to the differences (Figure 2D; p<0.003). Analysis of primary latency for individual trials revealed significant differences between the control and radiated groups on trial 4 of day 1 (Figure 2E; p = 0.0002) as well as trial 1 and 4 on day 2 (p<0.003). Both groups were able to recall the location of the target box during the Probe trial on day 5 (data not shown) and day 10 (Figure 2F). Taken together, these results demonstrate impairment in the rate of learning following WBRT since the ability to learn the task is maintained and deficits in learning acquisition occur during the first 2 days of training.

### Differences in aspects of working memory at 4 months following WBRT

**Figure 3:**
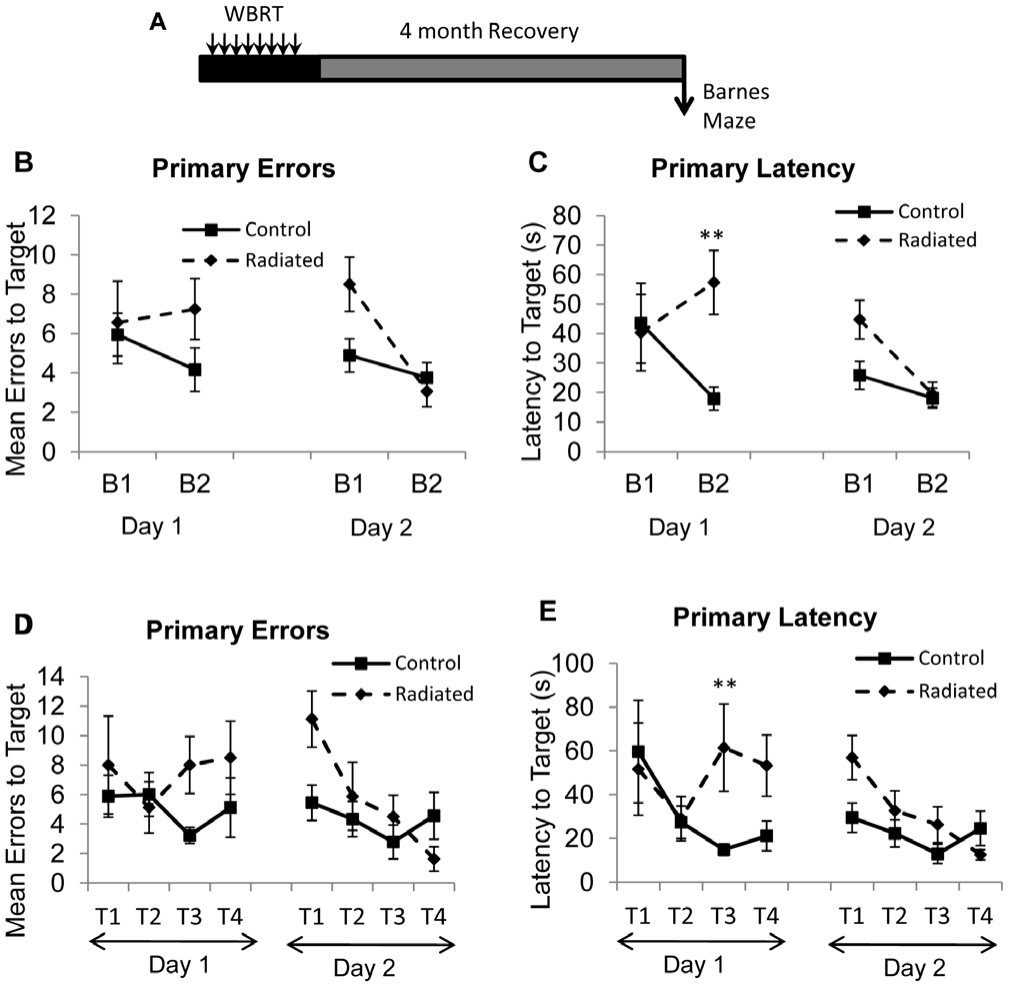
Working memory deficits are evident in radiated mice 4 months post-WBRT. (A) Schematic of the experimental design used. Impairments in learning occur in the radiated group as measured by: (B) primary errors and (C) primary latency over blocks of trials. Performance during individual trials is shown in (D) as primary errors and (E) as primary latency. Data represent Mean ± SEM. **p<0.00625 vs. Control. N = 9 animals per group.

Mice were tested on the working memory version of the Barnes maze to determine whether working memory is affected by WBRT. Four months after completion of fractionated WBRT (Figure 3A), significant radiation-induced deficits in learning acquisition were observed. Animals in the radiated group made more errors [F(1, 120) = 4.87; p = 0.0293] and took longer to locate the target hole compared to the controls [F(1, 120) = 5.75; p = 0.0181] (data not shown). Analysis of data represented as blocks of trials (2 trials per block, Figure 3B, 3C), revealed significant differences between control and radiated groups in latency to locate the target hole (Group × Day × Block) [F(2, 60)  = 3.5; p = 0.0365] while the number of errors made over blocks approached significance [F(2, 60)  = 2.6; p = 0.083]. There was a trend for increased Primary Errors in the radiated group on block 2 of day 1 and block 1 of day 2 (Figure 3B) and a significant increase in Primary Latency on block 2 of day 1 (Figure 3C; p = 0.0016). Further analysis of the individual trials per day (Group × Day × Trial) showed no significant differences between groups for primary errors (Figure 3D) although there was a trend for increased errors on trial 1 of day 2 (p = 0.0232). Radiated animals required more time to locate the target hole on trial 3 of day 1 (Figure 3E; p = 0.0054). These data demonstrate that specific aspects of working memory are impaired following WBRT.

### Learning acquisition and memory retention are impaired at 5 months post-WBRT

**Figure 4:**
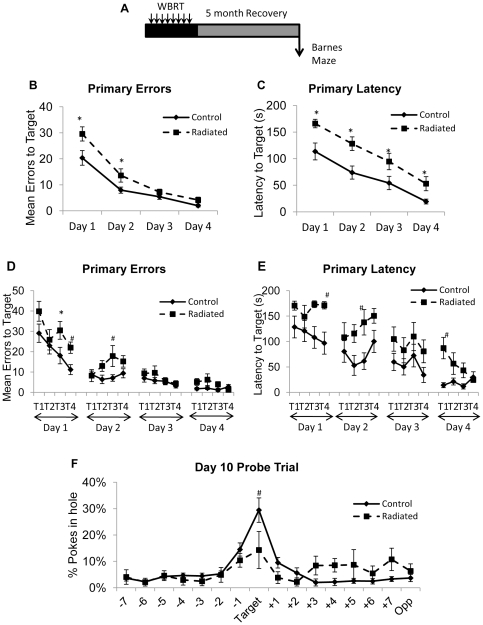
Radiated animals are impaired in both learning and memory retention at 5 months post-treatment. A schematic of the experimental design is shown in (A). Impairments in acquisition of the task, indicated by increased primary errors (B) and primary latencies (C) are observed in the radiated group. Representation of primary errors (D) and primary latency (E) over individual trials. (F) Long-term learning retention is impaired in the radiated group. Data represent Mean ± SEM. *p<0.0031; ^#^p<0.006 vs. controls. N = 8–10 animals per group.

To determine whether cognitive deficits induced by WBRT are maintained over several months, mice were tested on the Barnes maze at 5 months post-WBRT (Figure 4A). There was a significant overall effect of Day of training [F(3, 256) = 100.91; p<0.0001] and Trial [F(3, 256) = 4.53; p = 0.0041] on primary errors, observed as improved performance, and a significant overall effect of Group [F(1, 256) = 24.07; (p<0.0001)]. There was also a significant Group × Day interaction between [F(3, 256) = 3.24; p = 0.0227] for Primary Errors. Animals in the radiated group demonstrated learning impairments, making more errors on day 1 and day 2 of training compared to the controls (Figure 4B; p<0.004 each).

Analysis of primary latency also revealed significant Group [F(1, 256) = 47.37; p<0.0001] and Day [F(3, 256) = 44.34; p<0.0001] effects. Although there were no significant interactions between Group × Day [F(3, 256) = 0.71; p = 0.6369] or Group × Trial [F(3, 256) = 0.57; p = 0.8952], individual comparisons within Days indicated that radiated animals required a longer time to locate the target box each day of training (Figure 4C; p<0.0125). Analysis of individual trials on each day revealed significant differences in primary errors on day 1, trial 3 (Figure 4D; p = 0.0014) and approached significance on trial 4 of day 1 and trial 3 of day 2. There was a trend for increased primary latency on trial 4 of day 1, trial 3 of day 2, and trial 1 of day 4 (Figure 4E) in the radiated group. Short-term memory (assessed 24h after the last training trial using a Probe trial) revealed no differences (data not shown). However, radiated animals exhibited impaired long-term memory on the Probe trial assessed on day 10 (Figure 4F). Radiated animals made fewer head pokes in the hole where the target box was located compared to the controls (29±5% vs. 14±7%; p = 0.002).

### Learning deficits are reversed by systemic hypoxia

**Figure 5:**
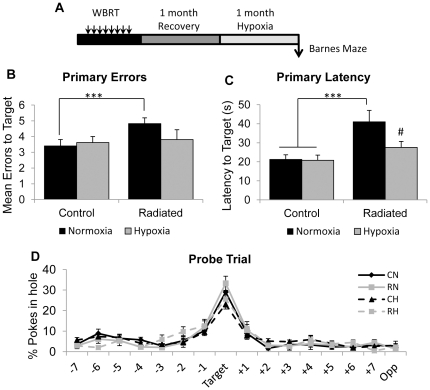
Systemic hypoxia completely reverses WBRT-induced learning deficits. A schematic of the experimental design is shown in (A). Radiated normoxic animals make more errors (B) and take longer to locate the target hole (C). These impairments are reversed in the radiated hypoxic group. (D) Learning retention is preserved in all groups. Data represent Mean ± SEM. ***p<0.0083 vs. Control Normoxic and ^#^p<0.0083 vs. Radiated Normoxic. N = 9 animals per group/treatment.

To determine whether systemic hypoxia can reverse WBRT-induced cognitive impairments, animals were subjected to 1 month of hypoxia (11% oxygen) initiated one month after the completion of WBRT (Figure 5A). Animals were re-acclimated to ambient oxygen conditions 48h prior to behavioral testing. The Barnes maze was used to assess learning and memory in response to WBRT and hypoxia treatment. The radiated group made significantly more primary errors [F(1, 512) = 5.09; p = 0.0245] and required a longer time [F(1, 512) = 28.01; p<0.0001] to locate the target hole. There was a significant overall effect of hypoxia on primary latency [F(1, 512) = 7.61; p = 0.006] but not primary errors [F(1, 512) = 1.23; p = 0.2680]. Radiated normoxic animals made more errors (Figure 5B) compared to the non-radiated normoxic group (4.8±0.4 vs. 3.4±0.4; p = 0.0052) whereas systemic hypoxia was able to abrogate differences in performance (3.8±0.6; p = 0. 67 compared to the control normoxic group). Radiated normoxic animals exhibited increased latency to locate the target compared to the control normoxic group (Figure 5C; 41.0±6.0s vs. 21.2±2.4s; p<0.0001) and systemic hypoxia was able to completely reverse these learning impairments (27.6±3.1s; p = 0.0002 compared to radiated normoxic group). No differences in long-term memory were detected (Figure 5D).

### Learning improvements induced by hypoxia are sustained after re-exposure to normal ambient oxygen levels

**Figure 6:**
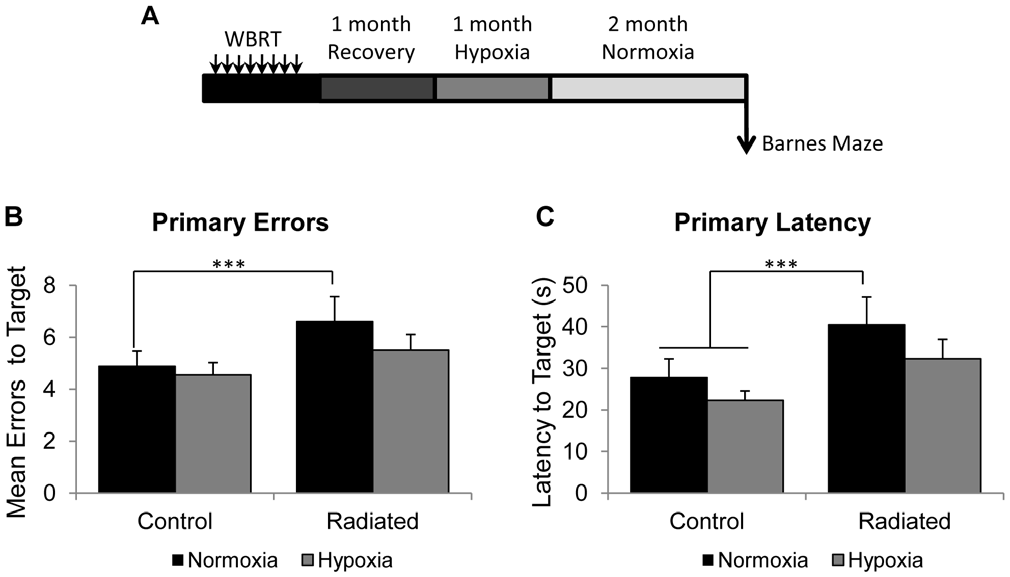
Benefits of systemic hypoxia are maintained after re-exposure to normal air. A schematic of the experimental design is shown in (A). Impairments in learning, indicated by increased primary errors (B) and primary latencies (C) are observed in the radiated normoxic group. Animals that were radiated and exposed to systemic hypoxia perform similar to the controls. Data represent Mean ± SEM. ***p<0.0083 vs. Control Normoxic. N = 8–9 animals per group/treatment.

Since our results indicated that hypoxia can reverse learning deficits induced by WBRT, we assessed whether the hypoxia-induced learning improvements are maintained after animals are returned to normal ambient oxygen levels (Figure 6A). The working memory version of the Barnes maze was used to assess memory. On day 1, the target box was placed in the same position as the last training session and on day 2, the target box was relocated to a new, random location. Mice were allowed 4 trials per day and primary errors and primary latency were recorded. Radiation had a significant effect on primary errors [F(1, 248) = 11.41; p = 0.0008] and primary latency [F(1, 248) = 6.62; p = 0.0106]. Significant impairments in learning were observed in the radiated normoxic animals evidenced by increased number of errors (Figure 6B, 6.6±1.0 vs. 4.9±0.6; p = 0.0174) and increased latency to find the location of the target box compared to the non-radiated normoxic group (Figure 6C, 40.4±4.5s vs. 27.8±4.5s; p = 0.0068). No differences were observed in the number of errors or latency for the radiated animals, previously treated with hypoxia, to locate the target box (p>0.05 compared to controls), demonstrating that the improvements in learning induced by systemic hypoxia were maintained for at least 2 months post-hypoxia.

### Recovery of vascular density after radiation and hypoxia is maintained after re-exposure to ambient oxygen

**Figure 7:**
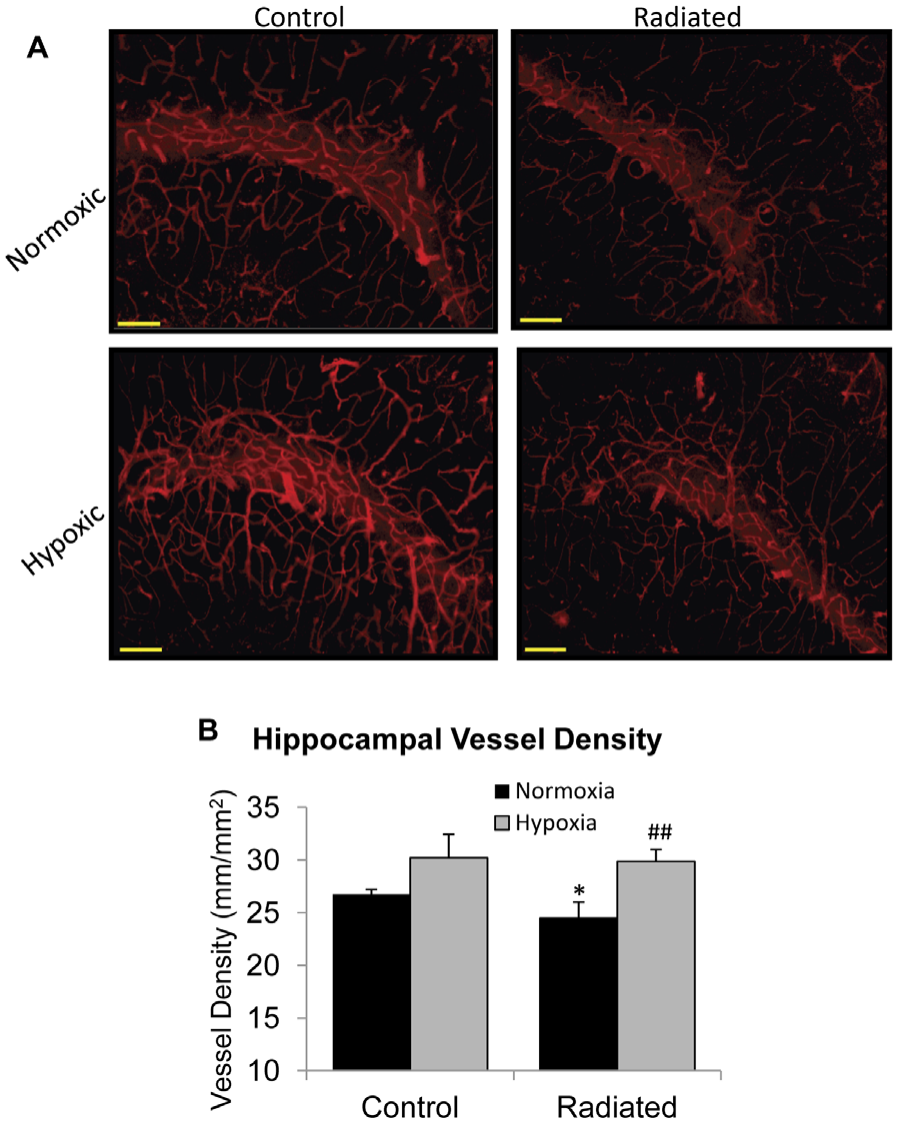
Changes in hippocampal vascular density after re-exposure to ambient oxygen levels. (A) Representative images of vascular density in the mouse hippocampus. Images were captured using fluorescence microscope at 10× magnification. Scale bar represents 100 µm. (B) Quantification of vascular density (length of vessels per area of tissue). Data represent Mean ± SEM. *p<0.05 vs. Control Normoxic and ^##^p<0.01 vs. Radiated Normoxic. N = 5–6 animals per group/treatment.

Hippocampal vascular density was quantified in the cohort of mice that were radiated, subjected to systemic hypoxia, and returned to normal ambient oxygen levels (Figure 6A) to determine whether the recovery of vascular density was maintained following re-exposure to normal air. There was a reduction in vessel density in the radiated normoxic group compared to the control normoxic group (24.47±1.53 mm/mm^2^ vs. 26.73±0.48 mm/mm^2^) (p = 0.0485; Figure 7A and B). Exposure to systemic hypoxia increased vessel density in the control (30.20±2.23 mm/mm^2^) and radiated animals for at least 2 months after discontinuation of the hypoxia (29.85±1.14 mm/mm^2^; p = 0.0051 compared to radiated normoxic group) demonstrating that the beneficial effects of were maintained.

## Discussion

In this study, we assessed the time-dependent changes in learning and memory that occur following fractionated WBRT using behavioral tests that assessed two cognitive domains (contextual and spatial learning). In addition, we investigated whether systemic hypoxia, shown previously to reverse WBRT-induced hippocampal capillary rarefaction, has the ability to increase cognitive function following a clinically relevant regimen of fractionated WBRT. Our results indicate that WBRT induces an early, transient decline in contextual learning and memory, impairments in working memory, and a progressive deterioration of spatial learning in mice. Consistent with our hypothesis that vascular rarefaction is associated with cognitive dysfunction, we show significant improvements in learning in response to chronic hypoxia, and demonstrate that these effects are maintained for at least 2 months after returning to normal ambient oxygen levels. Sustained behavioral benefits of systemic hypoxia are accompanied by sustained improvements in hippocampal microvascular density. This is the first study, to our knowledge, that presents a detailed time-course for WBRT-induced cognitive impairments in mice and demonstrates that systemic hypoxia can reverse learning deficits induced by WBRT. Our data provide critical support for the hypothesis that microvascular rarefaction is a key component of the deficits in learning and memory that occur after WBRT and that radiation-induced cognitive impairment can be reversed by appropriate treatment.

Cognitive dysfunction is a well-documented and established consequence of WBRT in both human and animal models. Clinical studies have shown that WBRT, alone or in combination with stereotactic radiosurgery leads to a significant decline in learning and memory 4–36 months [17], [18] following treatment even in the absence of tumor recurrence. Other studies of cognitive function after WBRT report reduced performance on tests of working memory [5], verbal memory [6], and general IQ [19]. Cognitive decline also occurs in animal models in response to brain irradiation. Liu et al. (2010) reported a dose- and time-dependent decrease in cognitive function in rats that was attributed to increased brain edema and disruption of blood-brain barrier function [20]. Impaired performance on the water maze has been reported to occur in rats in response to both single [21] or fractionated [8] whole brain radiation one year following treatment. In addition, deficits in spatial learning using the water maze [9] and Barnes maze [22] have been reported to occur in mouse models. Despite the reported effects of WBRT on cognitive function, a time-course for the appearance of cognitive dysfunction has not been reported in mice and the effects on specific cognitive domains has not been investigated. In this study, we show time-dependent impairments in both spatial and contextual learning in mice following fractionated WBRT. Interestingly, deficits in contextual learning appeared early and dissipated within 3 months after completion of the fractionated series of radiation while deficits in spatial learning were first evident at 2 months post-radiation and were maintained. Although significant differences in spatial learning were observed at both 2 and 5 months post-radiation, radiated animals were capable of acquiring the task at both time points. At 2 months post-radiation, deficits in errors and latency were evident on days 1 and 2 of training, which was characterized by sporadic performance of these animals on Trial 4 of day 1 (latency) and trial 3 and 4 of day 2 (for both primary errors and latency), demonstrating that the rate of learning in the radiated animals was impaired. No memory deficits were evident at 2 months post-radiation on the day 10 probe trial. By 5 months post-radiation, performance of radiated animals demonstrated more consistent impairments in acquisition across days (day 1 and 2 for primary errors and days 1–4 for primary latency) although both groups were able to learn the task. These results suggest that a more challenging spatial memory task may be able to separate radiated from non-radiated animals at these time points. Nevertheless, in contrast to the lack of differences in memory performance 2 month post-radiation, clear differences were evident in the day 10 probe trial at 5 months post-radiation. These data provide compelling evidence that there is a progressive deficit in spatial memory after WBRT and that specific cognitive domains are differentially influenced by WBRT. Furthermore, these data support the conclusion that there may be unique biological mechanisms that contribute to the impairments in learning and memory for each of these cognitive domains.

It is well-known that neurons are highly metabolic cells that require a controlled, steady supply of nutrients, oxygen, and growth factors, and efficient removal of carbon dioxide and metabolic wastes. In order for these functions to occur successfully, an intact neurovascular network is required. The neurovascular unit comprises of neurons, astrocytes, pericytes, and endothelial cells of the blood vessel wall [23]. Initial studies by Brown (Brown et al 2005) indicated that fractionated WBRT resulted in a generalized vascular rarefaction in the brain. These studies were later expanded and it was found that capillary rarefaction occurred in hippocampal sub-regions associated with spatial learning and memory [16]. These studies also reported the presence of chronic tissue hypoxia in these brain regions suggesting a failure of angiogenic mechanisms after the initial radiation-induced endothelial apoptosis. This conclusion is supported by studies demonstrating decreased angiogenesis after radiation [24]. Importantly, cerebrovascular rarefaction, associated with loss of both endothelial cells and pericytes [16], has been reported to precede cognitive deficits [7]. Additionally, in other models, cerebrovascular rarefaction has been strongly associated with cognitive impairments [25] and recovery of vascular density improves cognitive performance [26]. Our current data, together with studies of vascular rarefaction after radiation, demonstrate that vascular loss precedes the progressive spatial impairments that appear following radiation. Notably, improvements in spatial learning in this study occur within the same timeframe as restoration of hippocampal capillary density after systemic hypoxia [16] strongly supporting the integral role of vascular density in the maintenance of neuronal function.

Although the specific mechanisms by which systemic hypoxia induce recovery of spatial learning are still under investigation, it is well-known that hypoxia stimulates angiogenic mechanisms in multiple tissues, including brain [16], [27]–[29]. Angiogenesis is a multi-step process that results in the formation of new blood vessels from pre-existing vascular networks [30]. In conditions of low oxygen, hypoxia-inducible factor-1 alpha (HIF-1α) is stabilized and activates downstream angiogenic factors including but not limited to vascular endothelial growth factor (VEGF) and erythropoietin [31]–[33]. Interestingly, we have reported that HIF-1α and VEGFR2 are increased in brain tissue after WBRT suggesting that there are no deficits in the capacity of the brain to detect the hypoperfusion and decreased oxygen levels. However, angiogenic mechanisms are not induced in the radiated animals without additional stimuli. Although the specific mechanism for this effect is unknown, we propose that WBRT may eliminate key tissue resident stem cells [34] or support cells necessary for localized angiogenic mechanisms.

Although the etiology of radiation-induced cognitive dysfunction is poorly understood, early studies of cognitive deficits were attributed to demyelination and necrosis [21]. However, these studies generally used high single doses of radiation and necrosis was observed after extended recovery. Studies of the effects of more clinically relevant fractionated doses of radiation indicate that the number of neurons and hippocampal volume are unaffected [35]. Nevertheless, changes in hippocampal NMDA subunits occur [8] indicating that synaptic mechanisms important for learning and memory are affected by WBRT. Deficits in spatial learning have also been attributed to decreased neurogenesis [9] and chronic inflammation assessed by changes in activated microglia and infiltrating peripheral monocytes [22]. Additionally, acute neuro-inflammation in the form of increased gene expression of pro-inflammatory cytokines in the rodent hippocampus have been suggested to contribute to cognitive impairments [10]. Increases in several cytokines and chemokines occur within 4 to 8h post-radiation but these increases are transient and expression decreases over several hours or days. Nevertheless, studies conducted in our laboratory could not identify changes in an array of inflammatory cytokines at the protein level 2 months post-radiation (unpublished data). Although we cannot completely eliminate latent inflammatory responses as a potential mechanism for cognitive dysfunction, our data are more consistent with a model in which radiation-induced inflammatory changes contribute to impairments in contextual memory that resolve over time. This resolution of contextual learning occurs despite the vascular rarefaction that is prominent 2 months after WBRT. On the contrary, spatial memory deficits continue and are closely associated with decreased vascular density, decreased neurogenesis and alterations in synaptic architecture.

Interestingly, both chronic [36], [37] and intermittent [38] systemic hypoxia have been shown to stimulate neurogenesis in the hippocampus. However, evidence for the effects of systemic hypoxia on radiation-induced learning deficits in the adult mouse is lacking. Our findings that chronic hypoxia can reverse WBRT-induced cognitive impairments support the hypothesis that recovery of an angiogenic pathway and the associated increase in neurogenesis are essential components of recovery of learning and memory following radiation therapy. Contrary to our findings, there are several studies that report cognitive deficits as a consequence of exposure to hypoxic conditions [39]–[43]. However, in these studies, hypoxia was administered at a critical age of development [39] generally as a marked decrease in oxygen concentration (over a brief time period) or with hypoxia followed by re-oxygenation [40], [43]. While these studies are important, it is difficult to compare to our model that was a gradual decrease in oxygen levels.

Studies indicate that neurogenesis occurs in a vascular niche [44] and therefore, we find it highly probable that fractionated radiation impairs the capacity for both angiogenesis and neurogenesis by destroying vascular-associated stem cell precursors. We hypothesize that systemic hypoxia or other interventions that replace the stem cell population in the brain restore both angiogenic and neurogenic properties of the brain and facilitate cognitive recovery. Although this is an intriguing hypothesis, the capacity of systemic hypoxia to restore stem cells within the brain after radiation has not been established. This area of research remains an important future direction.

Recently, it has been reported that the decrease in neurogenesis after WBRT can be reversed by the introduction of human embryonic stem cells into the mouse hippocampus, resulting in improved cognitive function [45]. These findings provide *prima facie* evidence that radiation-induced decreases in neuronal stem cells and neurogenesis are important contributing factors to the cognitive deficits after WBRT. Importantly, previous data indicate that hippocampal neurogenesis is required for learning and memory [46], [47]. In the adult brain, neurogenesis encompasses the formation of new, functional neurons from neural precursor cells [48]. It is well established that brain radiation inhibits neurogenesis [49]–[55] suggesting that this decrease contributes directly to impairments in learning and memory [56]. Nevertheless, the transplantation studies did not analyze whether angiogenesis was restored in the radiated animals. Although this is an area that requires further investigation, we find it highly probable that both angiogenesis and neurogenesis are restored by cell transplantation suggesting that the effects of radiation on the brain are related to impairments in function of resident stem cells in the brain.

In summary, WBRT induces transient impairments in contextual learning, and a progressive deterioration of spatial learning. Systemic hypoxia, previously shown to reverse WBRT-induced hippocampal microvascular rarefaction, also completely reverses impairments in spatial learning. These data support the hypothesis that radiation-induced microvascular rarefaction and the corresponding impairments in spatial memory result, in part, from loss of angiogenic potential within the brain and that systemic hypoxia can reverse these deficits.

## Materials and Methods

### Animals

Ten (10) week-old C57BL/6 male mice were obtained from Charles River Laboratory (Wilmington, MA). Animals were maintained on a 12 hr light/dark cycle and fed standard rodent chow and water *ad libitum*. All animal protocols were approved by the Institutional Animal Care and Use Committee of the University of Oklahoma Health Sciences Center.

### Irradiation

Following acclimation to the animal facility for two weeks, mice were randomly assigned to control or radiated groups (Table 1). Animals were weighed and anesthetized *via* intra-peritoneal injection of ketamine and xylazine (100/15 mg per kg). Mice in the radiated group received a fractionated series of WBRT administered as 8 fractions of 4.5 Gy per fraction, twice a week for 4 weeks (total cumulative dose of 36 Gy), at a rate of 1.23 Gy/min while sham animals were anesthetized and not radiated. Radiation was administered using a ^137^Cs gamma irradiator (GammaCell 40, Nordion International). A Cerrobend® shield was utilized to minimize the dose to the bodies of the mice in the radiated group. Dosimetry was performed as described in [16] to confirm the dose received by the head and bodies of animals. Briefly, radiochromic films were strategically placed within the Cerrobend® shield to represent the anterior and posterior surfaces and the centers of the head and body of the mice. The irradiator was then activated for a time calculated to deliver 4.5 Gy to an empty irradiator chamber. Film analysis indicated that the body of the mice received an average of 1.06 ± 0.05 Gy, while the head received 4.4 ± 0.2 Gy.

**Table 1:** Summary of the animals and behavioral tasks used for this study.

Time post-WBRT (months)	Number of Mice per group	Behavioral Task
1	16–17	Active Avoidance
2	9	Barnes Maze
3	10–11	Active Avoidance
4	8–9	Barnes Maze
5	8–10	Barnes Maze

Mice were behaviorally tested using the active avoidance task and Barnes maze at 1, 2, 3, 4, and 5 months post-WBRT.

### Hypoxia Treatment

One month following sham or WBRT, mice were further divided into normoxic (21% oxygen) or hypoxic (11% oxygen) conditions. Hypoxia was achieved through the use of a custom-designed Plexiglass chamber (40.5 in ×24 in ×10.5 in), capable of holding 6 mouse cages. Air flow through the chamber was provided by compressed air and holes drilled into the individual mouse cages. Nitrogen gas and room air were regulated to create a final ambient oxygen level of 11% inside the chamber. Hypoxia was induced gradually by reducing the levels of oxygen by 1.5% daily, until the target level of 11% oxygen was obtained, after which, oxygen levels were maintained at 11% from day 7 through day 28. Oxygen levels were monitored at least twice daily using an oxygen meter (Extech Instruments, Waltham, MA) inserted through a fitted port. Adjustments to gas flow were made as necessary. Cage changes were performed weekly, exposing animals to room air for short periods (no more than 5 minutes). Animals in the normoxic group were housed in similar cages as hypoxic animals with oxygen levels maintained at 21%.

### Active Avoidance

One month and three months following sham or WBRT, mice were behaviorally characterized using an active avoidance task to determine cognitive status following treatment. Briefly, the active avoidance apparatus is comprised of a light and dark chamber, separated by an electrically controlled door, and assesses the ability of an animal to associate a noxious stimulus (foot shock) with a specific environment (dark chamber). On training day, mice were placed in the dark compartment with the door closed. An inescapable shock of 2 mA was delivered for 2s after which the door to the light chamber was opened and latency to enter the light side was recorded. Training was repeated after 10s and the mouse was returned to its home-cage. During the test periods (45 min and 24 h after training), the mouse was placed in the dark compartment, the door opened, and latency to enter the light chamber (in the absence of the shock stimulus) was recorded.

### Barnes Maze

The Barnes maze is a dry version of the water maze, designed to alleviate the stress of swimming [57]. Training and probe tests were performed as described [58] with minor modifications. Briefly, a sixteen-hole, circular maze was utilized to assess spatial learning following WBRT and systemic hypoxia. Mice were acclimated to the escape box (hidden beneath one of the holes) 24 h before training was initiated. Animals were then subjected to 4 trials per day with an inter-trial interval of 20 min over 4 consecutive days. The number of head pokes in incorrect holes (primary errors) and the latency to locate the target hole (primary latency) were recorded. Animals relied on spatial cues posted on the wall of the maze for orientation in order to escape the aversive stimuli of white noise and bright light. To assess memory, a probe trial was performed on day 5 (24 h after the last day of training) and day 10 (long-term memory). The probe trial is used to assess memory recall. The escape box is removed and the number of head pokes into each hole is counted within a 90s time-frame. If the animals recall the previous location of the escape box, it is expected that the number of head pokes at that location would increase.

### Working Memory Version of the Barnes Maze

To avoid carry-over of residual memory from previous training, a working memory version of the Barnes maze was used as described [59] with minor modifications. Briefly, mice were trained on the Barnes maze (described above) and 2 months after the last training trial, a working memory task was administered. The mice were tested for a total of 2 days (four trials per day). On day 1, the escape box was placed in the same location used during the last training session. On day 2, the location of the escape box was randomly moved to a different location. The number of errors and latency to the target hole were recorded.

### Vessel Density Analysis

Following completion of the working memory task, mice were anesthetized using ketamine/xylazine (100/15 mg/kg) via intramuscular injection. Mice were then transcardially perfused using Phosphate Buffered Saline, brains removed, hemisected, and left hemispheres post-fixed overnight in 4% paraformaldehyde. Post-fixed left hemispheres were cryoprotected in a series of graded sucrose solutions (10%, 20%, and 30% overnight each), and frozen in Cryo-Gel (Electron Microscopy Sciences, Hatfield, PA) for sectioning. Coronal sections of 70 µm were cut through the hippocampus and stored free-floating in cryoprotectant solution (25% glycerol, 25% ethylene glycol, 25% 0.1M phosphate buffer, 25% water) at −20°C. Sections selected for vessel density analysis were standardized using common anatomical planes following the Comparative Cytoarchitectonic Atlas of the C57BL/6 and 129/Sv Mouse Brains [60]. Selected sections were approximately 1.6mm caudal to Bregma, representing the more rostral hippocampus. After blocking in 5% BSA/TBS (with 0.5% Triton X-100) for 1 h, sections were immunostained using antibody against mouse CD31 (1: 100, phycoerythrin (PE) conjugated; BD Pharmingen; San Jose CA) overnight at 4°C. Sections were washed for 10 minutes in TBS (×6), transferred to slides and coverslipped. Images were captured using fluorescence microscopy (Nikon,) using a 10× objective. Capillary density in the stratum lacunosum moleculare (SLM) of the hippocampus (shown in Figure 7A) was quantified as the length of blood vessels per area of tissue using Neurolucida with AutoNeuron (MicroBrightField, Williston, VT). The total length of capillaries (mm) was divided by the area of hippocampal tissue (mm^2^) to obtain capillary density (length per area of tissue). The experimenter was blinded to the groups and treatments of the animals throughout the period of blood vessel staining and analysis.

### Statistical Analysis

Differences in performance on both the active avoidance task and the Barnes maze were analyzed using a within subjects Group × Day × Trial repeated measures analysis of variance (SAS version 9.2, SAS Institute, Cary, NC). The effects of systemic hypoxia on cognition were analyzed using Group × Condition (normoxic or hypoxic) × Day × Trial repeated measures analysis of variance. For post-hoc analysis, the Bonferroni t-test was used to determine differences between groups based on *a priori* hypotheses. Data are represented as mean ± standard error of the mean (SEM) with a statistically significant difference set at p<0.05.
